# Eukaryotic origins

**DOI:** 10.1098/rstb.2014.0321

**Published:** 2015-09-26

**Authors:** James A. Lake

**Affiliations:** MCDB Biology and Human Genetics, University of California, 232 Boyer Hall, Los Angeles, CA 90095, USA

**Keywords:** eukaryotes, eocytes, evolution, origin, dawn cell, nucleus

## Abstract

The origin of the eukaryotes is a fundamental scientific question that for over 30 years has generated a spirited debate between the competing Archaea (or three domains) tree and the eocyte tree. As eukaryotes ourselves, humans have a personal interest in our origins. Eukaryotes contain their defining organelle, the nucleus, after which they are named. They have a complex evolutionary history, over time acquiring multiple organelles, including mitochondria, chloroplasts, smooth and rough endoplasmic reticula, and other organelles all of which may hint at their origins. It is the evolutionary history of the nucleus and their other organelles that have intrigued molecular evolutionists, myself included, for the past 30 years and which continues to hold our interest as increasingly compelling evidence favours the eocyte tree. As with any orthodoxy, it takes time to embrace new concepts and techniques.

## 1. From ribosome structures to genes and genomes: the evolution of the eocyte tree

In 1983–1984, Walter Fitch walked into my UCLA office during his sabbatical. His visit changed my scientific life. My laboratory was reconstructing ribosome structures, mapping the locations of their proteins and rRNAs using immunoelectron microscopy, and growing the first three-dimensional crystals of ribosomal subunits [1]. I was intrigued by the unusual ribosomal substructures that we had found in an organism called *Sulfolobus solfataricus* and wanted to understand why the ribosomal substructures found in this prokaryote were very similar to those present in eukaryotes [2].

As I explained my ideas to Walter, he replied in his very direct way that I had it all wrong! But we continued our discussions over many weeks as he taught me how to use parsimony, his favourite method for analysing evolutionary trees. In retrospect, our exciting, collegial arguments gave me a conceptual understanding of evolution that would soon allow us to infer the deep eocyte, i.e. dawn cell, roots of eukaryotes from ribosome structures [3], from gene sequences, and ultimately from genomes.

**Figure 1.**
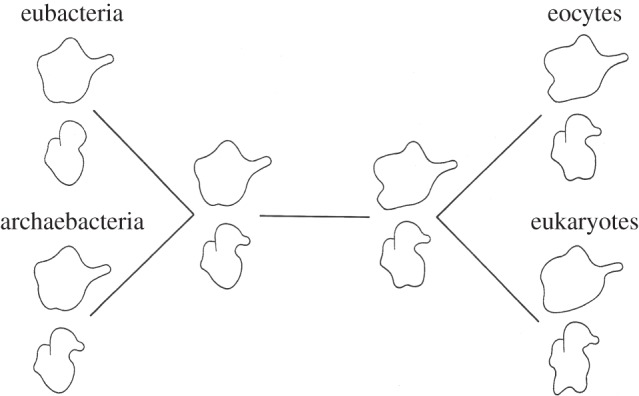
This first ‘eocyte tree’ was reconstructed based on the presence and absence of two ribosomal substructures. These substructures, an additional basal small subunit lobe and an additional lateral large subunit lobe are present exclusively in eukaryotes and in eocytes, and absent in ‘eubacteria’ and ‘archaebacteria’. Both substructures most parsimoniously support the eocyte tree. Note that this is an unrooted tree. Adapted from [3].

**Figure 2.**
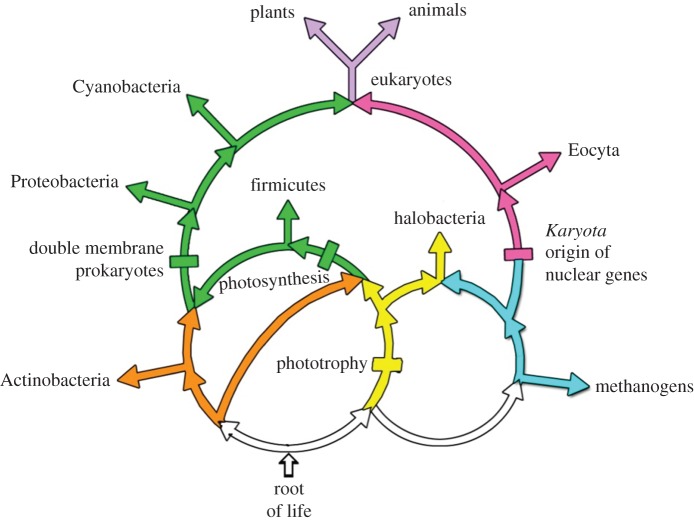
The sister group relationship of the eocytes to the eukaryotes is illustrated by the magenta ‘informational gene flow’ shown on the upper right side of the rings of life. It starts at the rectangle marked ‘Karyota’ and bifurcates to the left to enter the eukaryotes (lavender) and to the right to enter the *Eocyta*. The *Eukaryota* and the *Eocyta* are sister taxa and together form the taxon named the *Karyota*. Formally, the *Eocyta* is the sister taxon to the eukaryotic ‘informational genes' [5] and the *Karyota* is the clade that includes the *Eukaryota*, the *Eocyta*, and their most recent common ancestor [6].

Our first study analysed three-dimensional ribosomal substructures using parsimony. Because ribosomal substructures evolve much more slowly than gene sequences, we circumvented the long branch attraction (LBA) artefact that can easily confound phylogenetic analyses based upon molecular sequences [4]. That first unrooted eocyte tree (figure 1) based on a single eocyte species, *S. solfataricus,* is still consistent with the rooted trees and rings being derived from gene sequences (figure 2). Currently, four phyla have been discovered/named within the *Eocyta*: the *Aigarchaeota* [7], *Crenarchaeota* [8], *Korarchaeota* [9] and *Thaumarchaeota* [10], as summarized in reference [11].

## 2. Reconstructing the origin of eukaryotes

At the inception of gene sequencing discovering, the origin of eukaryotes was a major scientific goal. Parsimony and distance approaches were the main methods in use, and very few scientists were aware that these simple methods were vulnerable to error when sequences evolved rapidly.

When parsimony fails, it does so in a recognizable fashion. LBA groups all of the slowly evolving sequences into a ‘slow-clade’ and all of the rapidly evolving sequences into a ‘fast-clade’. The initial attraction to parsimony when genes were first being sequenced was that it appeared to provide strong, often suspiciously strong, support for LBA trees. In the early days of phylogenetic reconstruction, parsimony's super strong, but incorrect, results made it the favourite algorithm for studying ‘deep phylogenies', and even today, LBA-sensitive algorithms are still being used and generating incorrect results.

When eukaryotic 18S ribosomal sequences were first being sequenced, a ferocious competition arose to discover the oldest eukaryote. During this period, every newly sequenced nuclear ribosomal RNA gene was analysed and compared with those from prokaryotes in hopes of discovering the oldest, i.e. the deepest branching, eukaryotic lineage. Every few months, a new sequence analysis would report the discovery of the ‘oldest eukaryote’. The idea that eukaryotes might be very old was exciting and seemed to have strong support.

Ultimately, the race to find the oldest eukaryote collapsed under the weight of the LBA artefact. The final straw leading to the demise of the *Archezoa* occurred when the long-branch leading to the *Microsporidia* (spore-forming intracellular eukaryotic parasites) was nearly an order of magnitude longer than the other branches within the ‘crown group’ of the eukaryotes [12]. Once recognized, this plus the discovery that all *Archezoa* have or once had mitochondria [13] signalled the death knell for the *Archezoa*. But it would take several decades longer for it to be widely accepted that the three domains tree was also caused by LBA.

## 3. The accumulation of evidence for the eocyte tree over time

I was fortunate to learn about LBA early on because it focused our laboratory on reconstructing evolution in ways that would minimize the effects of LBA. Along the way, we developed several new analytical methods that used novel mathematical approaches to make them less affected by LBA. These included operator metrics, paralinear distances [14], closely related to LogDet which was independently discovered [15], and especially evolutionary parsimony [16] which is based on group theory and should not be confused with parsimony. All of these were more resistant to LBA than other contemporaneous methods. In 1988, evolutionary parsimony was used to reconstruct the ‘origin of the nucleus' [17]. That paper was widely covered by the press and it produced letters from around the world, some by anti-evolutionists. I still remember one from a witty fundamentalist who wrote, ‘ … you say humans came from an organism that lived at high temperature and smelled of sulfur. I have news for you, that's not where we came from, that's where you're going’.

Although the 1988 paper was quickly challenged [18], a few years later, I was extremely impressed to find that Manolo Gouy, the junior author of the paper that initially challenged eocytes, subsequently published analyses supporting the *eocyte* tree [19]. This result showed that future leaders in the field were beginning to change their minds as new data were collected and new methods developed. It also gave me hope that the technical details related to LBA were beginning to resonate within the phylogenetic reconstruction community.

In the beginning, the eocyte hypothesis had the support of several leading evolutionary biologists including: Walter Fitch (UC Irvine), Alan Wilson (UC Berkeley) and Colin Patterson (Natural History Museum, London). At that time, few biologists were familiar with the phylogenetic arguments against LBA, so my wife suggested that I apply for funding from the Sloan Foundation to hold winter schools on Evolutionary Biology at UCLA. Similar short courses offered by the MRC in Cambridge had shaped structural biology. I hoped that evolutionary short courses would provide the analytical skills to advance evolutionary biology. These distinguished evolutionists were extremely helpful in getting support from Sloan. The Sloan courses were highly successful and featured speakers such as Wally Gilbert, of DNA sequencing fame, the novelist Irving Stone (who wrote a biography of Darwin), Alan Wilson and Walter Fitch. They helped train a new generation of evolutionary biologists and many of our former students are now leaders in their fields.

Alan Wilson encouraged us to use PCR to sequence ribosomal RNAs and other informational genes from eukaryotes, potential eocytes and reference taxa. Thus, we sequenced many eocyte genes. Among the most useful genes that we sequenced were those coding for protein synthesis elongation factor EF-Tu, because it revealed the existence of an important indel (insertions and deletions within genes) that strongly supported the eocyte evolution of eukaryotes [20]. Even today, those results are so compelling to me that I still do not understand why they were not more widely accepted at the time.

The eocyte controversy also brought with it some unexpectedly positive benefits. It taught us how to quickly sequence genes using PCR, and it also forced us to develop new analytical methods that could handle LBA. Thus, we were positioned to sequence and analyse the relationships between major animal groups using a suite of new tools. The presence of LBA was quickly recognized but we then knew how to circumvent it. As a result we proposed the ‘new animal phylogeny’ that consists of the *Deuterostomia*, the *Lophotrochozoa* and the *Ecdysozoa* [21–23].

Without the eocyte controversy, we might never have discovered the new animal phylogeny, because our success depended upon being able to compensate for LBA. I will never forget the excitement when our evolutionary parsimony calculations first showed that the nematodes and the arthropods were sister taxa—nematodes were then thought to be Aschelminthes. I sat back in my chair almost in shock and suddenly realized that these sister taxa both moulted their exoskeletons, and then called my collaborators. For the first time, to the best of my knowledge, a multicellular animal tree had been reconstructed that directly related a genotype to a phenotype (moulting or ecdysis). And it was a huge animal group containing many more species than any other animal super phylum.

In many ways, the new animal phylogeny marked the start of a new phylogenetic era in which LBA was increasingly recognized as a major problem for phylogenetic reconstruction. Because our publications were in highly visible journals, they received much attention, but so did our first eocyte publications. Something was clearly happening in the field of multicellular animal evolution that was different from the first time around. For some reason, the new animal phylogeny met with little resistance, soon entered the textbooks, and was fully accepted by the 150th anniversary of the publication of the *Origin of Species*.

## 4. The beginnings of an evolutionary renaissance

The eocyte quest also took on some of the aspects of ‘six degrees of separation’. For example, my former graduate student, now Prof. Janet Sinsheimer, and her first graduate student, now Prof. Marc Suchard, developed sophisticated, continuous time Markov models in order to test the eocyte hypothesis [24]. Their methods were precursors to more recent approaches such as NDCH [25] and CAT [26] that by better modelling evolutionary processes led to the recent demonstrations of strong support for the eocyte phylogeny.

In 2008, my wife and I were in Hawaii on holiday when I got an email asking if I would review a manuscript for *PNAS* on the eocyte hypothesis. I was really excited by the abstract, but by the time I got back to the editor, another reviewer had signed on. To me that paper marked the beginning of the resurgence of the eocyte classification [27]. Since that time, the eocyte hypothesis has been recovered by phylogenetic analyses published by several laboratories using better methods [28–33], so that it is has now emerged as the consensus phylogenetic framework for understanding eukaryotic nuclear origins.

## 5. What are the remaining questions and challenges?

An outstanding challenge is how to relate the eocyte tree and other new findings to eukaryotic evolution more broadly. The eocyte hypothesis deals with the ancestry of the nuclear host lineage and eukaryotic informational genes, but those genes are only one part of the eukaryotic gene complement. Thus, it is clear from our own work [5] and that of others [34–36] that eukaryotic genomes contain many genes for metabolism that are mainly, but not exclusively, of bacterial ancestry. I have argued [6,37] that the chimeric nature of cellular genomes, prokaryotic as well as eukaryotic, can be best understood by a combination of large gene flows and cycle graphs to represent genomic mergers. Our current understanding and hypotheses for the evolution of eukaryotes based upon these ideas and analyses is summarized in figure 2. At least two gene flows merge to form the eukaryotes. These are the informational genes, shown in magenta on the right and the operational genes shown in green on the left [5]. The operational genes are present in eukaryotic chloroplasts and mitochondria, and the informational genes are present in the eukaryotic nucleus. The genes within the informational gene flow underpin the eocyte tree discussed above. The eocytes, formally the *Eocyta* (‘dawn cells'), and the eukaryotes are sister taxa within the eukaryotic informational gene flow as shown in the upper right part of figure 2. Together the eocytes, the eukaryotes and their last common ancestor form the taxonomic group known as the *Karyota* [38], or the karyotes informally. This sister group based definition of the *Eocyta* provides the phylogenetic basis for experimentally identifying additional eocytes, and suggests clues to the origin and evolution of the nucleus.

The operational gene flow shown in green at the upper left reflects its complex symbiotic origins. The operational gene flow is proposed to have supplied the eukaryotic mitochondria and chloroplasts, and is related to the complex acquisition of these and possibly other eukaryotic cytoplasmic organelles [13]. It is also related to the photosynthetic gene flow [39], shown in green, and to the earlier photrophic gene flow [40] shown in yellow. The rings are rooted at the bottom of figure 2, based on indels incompatible with other possible roots [6].

Other important challenges include studying the early evolution of eukaryotes and more accurately mapping the origins of their informational and operational genes. As we continue to learn more about eukaryotic evolution, we position ourselves to understand the evolution of developmental pathways, in order to relate them to human health, and to understand our evolutionary beginnings. The early evolution of eukaryotes has been complex, and I suspect that the early evolution of humans and other eukaryotes will be equally and possibly far more complex.

Many other major problems are waiting to be solved. Gene divergences and gene convergences of the sort that simultaneously determine both tree-like and ring-like evolution have much to tell us. They can inform us about the deep beginnings of prokaryotes and eukaryotes and they can do it in ways that that can potentially allow us to relate genotypes to phenotypes, but new, improved analytical methods will be needed to reconstruct ring-like evolution.

I am optimistic about the future of evolutionary phylogenomics, especially given the many improvements being made to reduce LBA. I believe that there may be an important story behind each of the gene flows within the rings of life, that those stories may be unlike any that we could have imagined in the past and may simultaneously lead to significant advances in improving human health. I predict that the story will only get better as we understand more about the evolution of life on the Earth. Enjoy the rest of this volume and as you read keep in mind the role of LBA.

As my first departmental chair, George Palade said to me upon his winning the Nobel prize, ‘It takes time for new paradigms to displace old orthodoxies, and the decision which is right has to be based on testing, and not on faith’.
